# Risk factors for early progression of diffuse low-grade glioma in adults

**DOI:** 10.1186/s41016-022-00295-z

**Published:** 2022-10-01

**Authors:** Long Wang, Xuegang Li, Tunan Chen, Chao Zhang, Jiantao Shi, Hua Feng, Fei Li

**Affiliations:** Department of Neurosurgery, Southwest Hospital, Third Military Medical University (Army Medical University), Chongqing, 400038 China

**Keywords:** Risk factors, Early progression, Low-grade glioma

## Abstract

**Background:**

To explore the risk factors for early progression of diffuse low-grade glioma in adults.

**Methods:**

A retrospective analysis of pathologic and clinical data of patients diagnosed with diffuse low-grade gliomas at Southwest Hospital between January 2010 and December 2014. The progression-free survival (PFS) less than 60 months was classified as the early progress group, and the PFS greater than 60 months was the control group for comparative analysis.

**Results:**

A total of 138 patients were included in this study, including 94 cases of astrocytoma and 44 cases of oligodendroglioma. There were 63 cases with 100% resection, 56 cases with 90–100% resection degree, and 19 cases with resection degree < 90%. The average follow-up time was 60 months, of which 80 patients progressed and 58 patients did not progress. The average progression-free survival was 61 months. The median progression-free survival was 60 months. There were 68 patients with *PFS* **≤** 60 months and 70 patients with *PFS* > 60 months. The two groups were compared for statistical analysis. In univariate analysis, there were significant differences in tumor subtype (*p* = 0.005), range (*p* = 0.011), volume (*p* = 0.005), location (*p* = 0.000), and extent of resection (*p* = 0.000). Multifactor analysis shows tumor location (*HR* = 4.549, 95% *CI*: 1.324–15.634, *p* = 0.016) and tumor subtype (*HR* = 3.347, 95% *CI* = 1.373–8.157, *p* = 0.008), and imcomplete resection is factors influencing early progression of low-grade glioma.

**Conclusions:**

Low-grade gliomas involving deep location such as basal ganglia, inner capsule, and corpus callosum are more likely to progress early, while incomplete resection is a risk factor in early progression of astrocytoma.

## Background

According to 2021 WHO new classification of gliomas, adult diffuse gliomas include IDH-mutant astrocytomas, oligodendrogliomas, and glioblastomas because of their shared driver mutations and similar growth patterns and biological behavior [1]. Diffuse low-grade glioma (DLGG) occurs frequently in young people and grows relatively slowly but almost always progresses into high-grade gliomas eventually [2, 3]. Previous studies show that low-grade diffuse gliomas progress into glioblastomas in 2 years to more than 10 years [4]. Most DLGGs progress slowly after surgery, but a small percentage of patients will progress rapidly. Currently, there is no reliable clinical imaging and pathological features to predict the early progression of DLGG. We retrospectively analyzed the clinical data of DLGG patients who underwent surgical treatment in 2010–2014, in order to investigate the risk factors related to early progress of DLGG.

## Methods

### Patient selection

In this study, we performed a retrospective analysis on adult patients with diffuse low-grade glioma who underwent surgery in our department and were followed up from January 2010 to December 2014. The cases complying with this study include WHO grade 2 astrocytoma, oligodendroglioma. For the patients younger than 18 years, those with low-grade gliomas of cerebellum, spinal cord, and brain stem were excluded. Progression-free survival (PFS) was defined as the time from the first surgery to T2-FLAIR follow-up showing abnormal signals or the date of last follow-up. We excluded patients with suspected pseudoprogression by extending the follow-up time. Imaging stability or disappearance was defined as pseudoprogression, and progressive growth was scored as progression. Patients with PFS less than 60 months were then classified as early progression group, and patients with PFS greater than 60 months were classified as control group.

### Data collection

Patient data were collected from our hospital’s electronic medical record system, including patients’s gender, age, preoperative tumor volume and location, first operation time, extent of resection (100%, 90–100%,< 90%), pathological type, postoperative treatment (radiotherapy alone, chemotherapy alone, radiotherapy + chemotherapy, no radiochemotherapy), and magnetic resonance images before surgery and in postoperative follow-up. We classified the tumors invading the inner capsule, basal ganglia, and corpus callosum as deep gliomas and the rest as superficial gliomas. Regarding the calculation of tumor volume: the maximum axial, coronal, and sagittal diameters of T2-FLAIR images were measured manually on the computer, and then, the tumor volume was calculated using the ellipsoid formula (*V* = 1/2 D1 × D2 × D3)). In terms of evaluation on the extent of resection, T2-FLAIR at 3 months after surgery was compared with T2-FLAIR tumor volume before surgery. Abnormal signal occurred on recently followed-up T2-FLAIR was defined endpoint event.

### Statistical analysis

Statistical analysis was carried out using IBM IPSS 25.0 software. Chi-square test or *T*-test analysis was performed for single factors in the early progress group and control group. Multiple logistic regression analysis was performed by using significant variables (*p* < 0.1) in the single factor analysis as candidate variables. Survival curve was plotted using Kaplan-Meier method, and survival difference was evaluated using log-rank test. Hazard ratio (HR) was evaluated with a 95% confidence interval (95% CI). The significance level was set at *p* < 0.05.

## Result

### Patient and treatment characteristics

A total of 694 glioma patients were admitted to our hospital from 2010 to 2014, among which 252 patients were diagnosed with WHO 2 and 43 patients were lost to follow-up. Moreover, patients younger than 18 years and those with subtentorial, spinal cord, and brainstem gliomas were excluded. Some patients did not undergo molecular testing ,and 26 patients diagnosed with oligoastrocytoma were excluded. Finally, a total of 138 patients were included as research objects, with a median follow-up time of 60 months (7–120 months). Sixty-eight patients with PFS are less than 60 months and 70 patients with PFS greater than 60 months. There were 94 patients (68.1%) with astrocytomas and 44 patients (31.9%) with oligodendroglioma. Sixty-two patients (44.9%) underwent total resection, 56 patients (40.6%) underwent subtotal resection, and 20 patients (14.5%) underwent partial resection. There were 107 cases (77.5%) of tumors in the superficial location and 31 cases (22.5%) of tumors in the deep location. After surgery, 80 patients underwent adjuvant radiotherapy, with a total dose of 54 Gy, 1.8 Gy each time, and five times a week, for totally 6 weeks; 85 patients underwent adjuvant chemotherapy, and the regimen included six courses of TMZ. After operation, 20 patients received radiotherapy alone, 25 patients received chemotherapy alone, 60 patients received radiotherapy combined with chemotherapy, and 33 patients did not receive radiotherapy and chemotherapy. Follow-up MRI of the head was carried out once every 6 months to 1 year. The median progression-free survival was 60 months. There were 68 cases with PFS ≤ 60 months and 70 cases with PFS > 60 months. See Table 1 for detailed clinical characteristics.

**Table 1:** Clinical and tumor characteristics of 138 patients

Variable	Recurrence (*n* = 80)	Non-recurrence (*n* = 58)
Sex, *n* (%)		
Male	39 (48.8)	28 (48.3)
Female	41 (51.2)	30 (51.7)
Age, years, *n* (%)		
≥ 40	38 (47.5)	28 (48.3)
< 40	42 (52.5)	30 (51.7)
Tumor subtype, *n* (%)		
Astrocytoma	59 (73.8)	35 (60.3)
Oligodendroglioma	21 (26.2)	23 (39.7)
Tumor location, *n* (%)		
Superfical	60 (75.0)	47 (81.0)
Deep	20 (25.0)	11 (19.0)
Tumor range, *n* (%)		
Single lobe	45 (56.3)	33 (56.9)
Multiple lobe	35 (43.7)	25 (43.1)
Preoperative tumor volume, cm^3^, *n* (%)		
≤ 30	26 (32.5)	29 (50.0)
> 30	54 (67.5)	29 (50.0)
EOR, *n* (%)		
100	36 (45.0)	26 (44.8)
90–100	32 (40.0)	24 (41.4)
< 90	12 (15.0)	8 (13.8)
Treatment after first surgery, *n* (%)		
RT alone	20 (25.0)	0 (0.0)
Chemotherapy alone	16 (20.0)	9 (15.5)
RT + chemotherapy	26 (32.5)	34 (58.6)
None	18 (22.5)	15 (25.9)

*EOR* extent of resection, *RT* radiotherapy

### Univariate and multivariate analyses related to progression-free survival

Comparative statistical analysis was performed between the control group (*PFS* > 60 months) and early progress group (*PFS* ≤ 60). Univariate analysis showed significant differences in tumor volume (*p* = 0.005), location (*p* = 0.000), subtype (*p* = 0.005), range (*p* = 0.011), and extent of resection (*p* = 0.000), but no significant differences between the two groups in gender, age, and postoperative treatment (Table 2). Multivariate analysis showed tumor location (*HR* = 4.750, 95% *CI*: 1.356–16.641, *p* = 0.015) and tumor subtype (*HR* = 3.508, 95% *CI* = 1.429–8.612, *p* = 0.006), and imcomplete resection is factors influencing early progression of low-grade glioma (Table 3).

**Table 2:** Univariate analysis for progression-free survival of early progress

Variable	PFS ≤ 60 months (*n* = 68)	PFS > 60 months (*n* = 70)	χ^2^/Z	*p*-Value
Sex, *n* (%)			0.113	0.737
Male	34 (50.0)	33 (47.1)		
Female	34 (50.0)	37 (52.9)		
Age, years, *n* (%)			0.269	0.604
≥ 40	31 (45.6)	35 (50.0)		
< 40	37 (54.4)	35 (50.0)		
Tumor subtype, *n* (%)			7.876	0.005
Astrocytoma	54 (79.4)	40 (57.1)		
Oligodendroglioma	14 (20.6)	30 (42.9)		
Tumor location, *n* (%)			12.370	0.000
Superfical	44 (64.7)	63 (90.0)		
Deep	24 (35.3)	7 (10.0)		
Tumor range, *n* (%)			6.521	0.011
Single lobe	31 (45.6)	47 (67.1)		
Multiple lobe	37 (54.4)	23 (32.9)		
Preoperative tumor volume, cm^3^, *n* (%)			7.938	0.005
≤ 30	19 (27.9)	36 (51.4)		
> 30	49 (72.1)	34 (48.6)		
EOR, *n* (%)			−4.529a	0.000
100	16 (23.5)	46 (65.7)		
90–99	38 (55.9)	18 (25.7)		
< 90	14 (20.6)	6 (8.6)		
Treatment after first surgery, *n* (%)			1.796	6.616
RT alone	10 (14.7)	10 (14.3)		
Chemotherapy alone	15 (22.1)	10 (14.3)		
RT + chemotherapy	29 (42.6)	31 (44.3)		
None	14 (20.6)	19 (27.1)		

*EOR* extent of resection, *RT* radiotherapy; ^a^nonparametric test

**Table 3:** Multivariate analysis for progression-free survival of early progress

Covariates	Exp (B)	95% *CI*	*p*-value
Tumor location, deep vs. superfical	4.750	1.356–16.641	0.015
Tumor subtype, astrocytoma vs. oligodendroglioma	3.508	1.429–8.612	0.006
Tumor range, multiple lobe vs. single lobe	0.667	0.233–1.913	0.451
Preoperative tumor volume, > 30 cm^3^ vs. ≤ 30 cm^3^	1.500	0.628–3.579	0.361
EOR			
100	1 [Reference]	1 [Reference]	
90–100	5.057	2.096–12.200	0.000
< 90	6.201	1.705–22.555	0.006

*EOR* extent of resection, *RT* radiotherapy

### Progression-free survival analysis

**Fig. 1:**
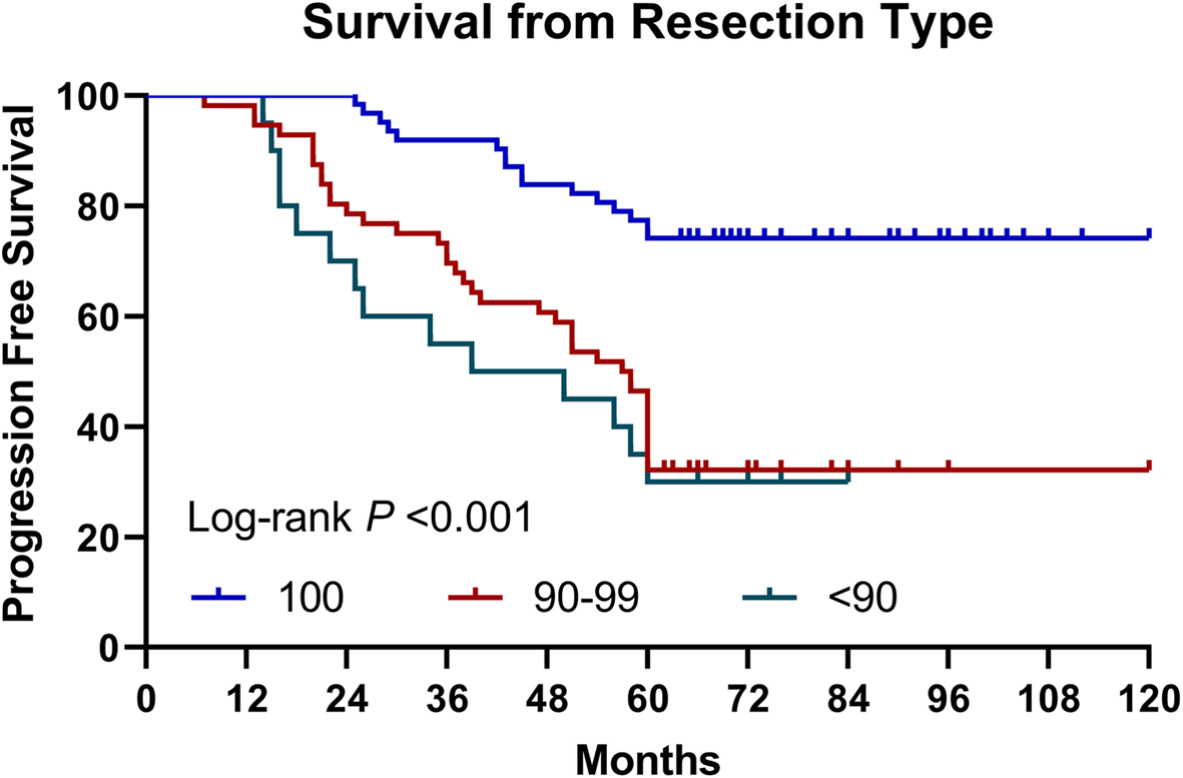
Progression-free survival curve of patients with different extent of resection (log rank *p* < 0.001). PFS, progression-free survival

**Fig. 2:**
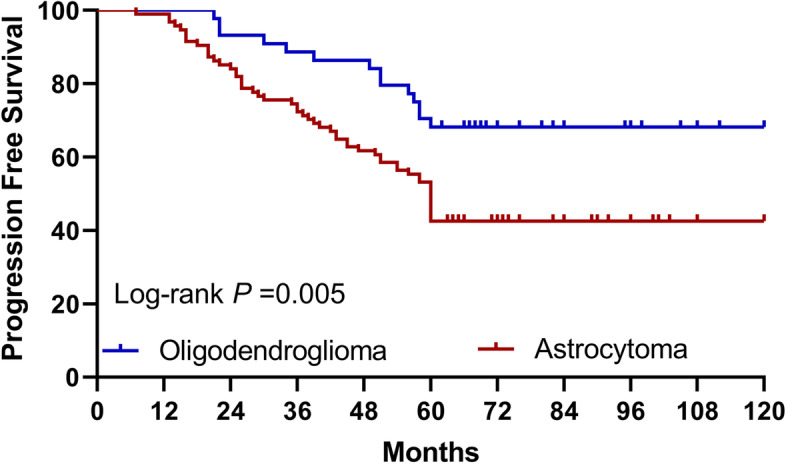
Progression-free survival curve of patients with different tumor subtypes (log rank *p* = 0.005). PFS, progression-free survival

**Fig. 3:**
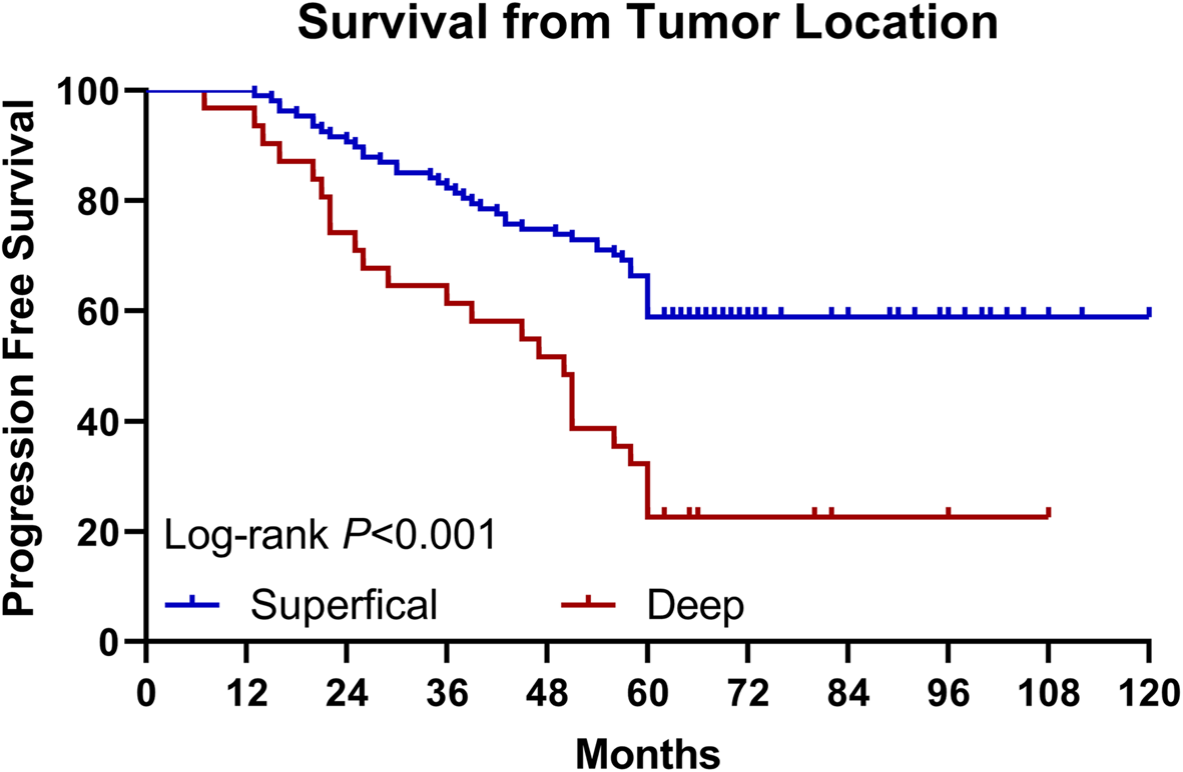
Progression-free survival curve of patients with different tumor location (log rank *p* < 0.001). PFS, progression-free survival

Kaplan-Meier was used to evaluate progression-free survival. The median PFS of patients with complete resection was 96 months, the median PFS of patients with 90–100% resection was 60 months, and the median PFS of patients with resection less than 90% was 39 months. The difference is statistically significant (*p* < 0.001) (Fig. 1). The median PFS of oligodendroglioma was 98 months, the median PFS of astrocytoma was 72 months, and the difference was considered statistically significant (*p* = 0.005) (Fig. 2). Patients with superficial tumors have a median progression-free survival of 92 months, and patients with deep tumors have a median progression-free survival of 50 months, with statistically significant difference shown (*p* < 0.001) (Fig. 3).

### Risk factors for early progression of astrocytoma

There were a total of 94 cases of astrocytoma, including 40 cases with 100% resection, 39 cases with 90–100% resection, and 15 cases with < 90% resection. After surgery, 54 cases underwent radiotherapy, and 62 cases underwent chemotherapy (see Table 4). The median progression-free survival was 60 months. There were 54 patients in the early progression group (*PFS* ≤ 60) and 40 patients in the control group (*PFS* > 60). Univariate analysis showed that there were significant differences in extent of resection (*p* = 0.005), tumor location (*p* = 0.001), preoperative tumor volume (*p* = 0.049), and tumor range (*p* = 0.034), which had no significant correlation with postoperative adjuvant radiotherapy and chemotherapy. Multivariate analysis showed that tumor location was the factor influencing early progression (Table 5).

**Table 4:** Univariate analysis of early progression of astrocytoma

Variable	PFS ≤ 60 months (*n* = 54)	PFS > 60 months (*n* = 40)	χ^2^/Z	*p*-Value
Sex, *n* (%)			1.154	0.283
Male	25 (46.3)	23 (57.5)		
Female	29 (53.7)	17 (42.5)		
Age, years, *n* (%)			0.797	0.372
≥ 40	32 (59.3)	20 (50.0)		
< 40	22 (40.7)	20 (50.0)		
Tumor location, *n* (%)			11.013	0.001
Superfical	36 (66.7)	38 (95.0)		
Deep	18 (33.3)	2 (5.0)		
Preoperative tumor volume, cm^3^, *n* (%)			3.871	0.049
≤ 30	34 (44.4)	15 (37.5)		
> 30	20 (55.6)	25 (62.5)		
Tumor range, *n* (%)			4.489	0.034
Single lobe	26 (48.1)	28 (70.0)		
Multiple lobe	28 (51.9)	12 (30.0)		
EOR, *n* (%)			−2.824^a^	0.005
100	16 (29.6)	24 (60.0)		
90–99	27 (50.0)	12 (30.0)		
< 90	11 (20.4)	4 (10.0)		
Treatment after first surgery, *n* (%)			3.862	0.277
RT alone	8 (14.8)	3 (7.5)		
Chemotherapy alone	13 (24.1)	6 (15.0)		
RT + chemotherapy	24 (44.4)	19 (47.5)		
None	9 (16.7)	12 (30.0)		

*EOR* extent of resection, *RT* radiotherapy; ^a^nonparametric test

**Table 5:** Multivariate analysis of early progression of astrocytoma

Covariates	Exp (B)	95% *CI*	*p-*value
Tumor location, deep vs. superfical	8.789	1.546–50.064	0.014
Tumor range, multiple lobe vs. single lobe	0.722	0.231–2.260	0.576
Preoperative tumor volume, > cm^3^ vs. ≤ cm^3^	1.518	0.567–4.064	0.406
EOR			
100	1 [Reference]	1 [Reference]	0.143
90–100	2.712	0.991–7.426	0.052
< 90	2.914	0.677–12.549	0.151

*EOR* extent of resection, *RT* radiotherapy

### Risk factors for early progression of oligodendrocytoma

There were a total of 44 cases of oligoglioma, including 22 cases of total resection, 17 cases of subtotal resection, and 5 cases of partial resection. From the perspective of tumor location, there were 33 cases with tumors located in the superficial part and 11 cases with tumor located in the deep part. There were 14 cases in the early progression group (*PFS* ≤ 60) and 30 cases in the control group (*PFS* > 60). Univariate analysis showed that there were significant differences in extent of resection (*p* = 0.000), revealing no significant differences in tumor location, tumor range, and preoperative volume (see Table 6 for details).

**Table 6:** Univariate analysis of early progression of oligodendroglioma

Variable	PFS ≤ 60 months (*n* = 14)	PFS > 60 months (n = 30)	χ^2^/Z	*p*-Value
Sex, *n* (%)			3.727	0.054
Male	9 (64.3)	10 (33.3)		
Female	5 (35.7)	20 (66.7)		
Age, years, *n* (%)			0.786	0.375
≥ 40	9 (64.3)	15 (50.0)		
< 40	5 (35.7)	15 (50.0)		
Tumor location, *n* (%)			2.235	0.135
Superfical	8 (57.1)	25 (83.3)		
Deep	6 (42.9)	5 (16.7)		
Preoperative tumor volume, cm^3^, *n* (%)			3.020	0.082
≤ 30	4 (28.6)	17 (56.7)		
> 30	10 (71.4)	13 (43.3)		
Tumor range, *n* (%)			2.937	0.087
Single lobe	5 (35.7)	19 (63.3)		
Multiple lobe	9 (64.3)	11 (36.7)		
EOR, *n* (%)			−4.239a	0.000
100	0 (0)	22 (73.3)		
90–99	11 (78.6)	6 (20.0)		
< 90	3 (21.4)	2 (6.7)		
Treatment after first surgery, *n* (%)			0.172	0.982
RT alone	3 (21.4)	7 (23.3)		
Chemotherapy alone	2 (14.3)	4 (13.4)		
RT + chemotherapy	5 (35.7)	12 (40.0)		
None	4 (28.6)	7 (23.3)		

*EOR* extent of resection, *RT* radiotherapy; ^a^nonparametric test

### Analysis of the relationship between tumor location and degree of resection

There were 107 cases of superficial tumor, including 60 cases with total resection, 36 cases with subtotal resection, and 11 cases with partial resection. In 31 cases of deep tumor, there were 7 cases with total resection, 17 cases with subtotal resection, and 7 cases with partial resection. The extent of resection of superficial tumor was significantly higher than that of deep tumor (χ^2^ = 11.153, *p* = 0.004). There were 78 cases of single lobe involvement, including 51 cases with total resection, 23 cases with subtotal resection, 4 cases with partial resection, and there were 60 cases of multiple lobes involvement, including 16 cases with total resection, 30 cases with subtotal resection, and 14 cases with partial resection. The extent of resection of single lobe was significantly higher than that of multiple lobes (χ^2^ = 22.804, *p* = 0.000).

## Discussion

Supratentorial low-grade diffuse gliomas in adult account for 15% of all gliomas [5]. DLGG is an aggressive, progressive, and chronic central nervous system disease. Such lesion grows sustainably, migrates along the white matter pathway, and inevitably develops into a high-grade malignant tumor, ultimately leading to death of the patient [5]. Two large-scale randomized studies by the European Organization for Cancer Research and Treatment (EORTC) show that the age is more than 40 years old, the tissue type is astrocytoma, the diameter is more than 6 cm, the tumor crosses the midline, and the preoperative neurological deficit is a risk factor for poor prognosis [6]. The survival time of DLGG patients varies greatly. The low-risk patients can survive for more than 10 years, while the high-risk patients normally only have 2 years [7]. Therefore, understanding the risk factors in the progression of diffuse low-grade gliomas is particularly important for preparing treatment strategies. In this study, we retrospectively analyzed the case data of 138 DLGG patients and statistically analyzed their progression-free survival to find relevant factors in early progression. The average follow-up time in this study was 60 months, which was relatively short, so we selected PFS as the endpoint event for the study rather than overall survival OS.

Diffuse low-grade gliomas are more common in young people, and an age over 40 is a risk factor for poor prognosis [6]. In this study, the early progress group was 18–72 years old, with a median age of 40 years old, while the control group was 18–56 years old, with a median age of 38 years old. There was a difference in age between the two groups, but such different was of no statistical significance. Early surgery and maximum safe resection are currently the recommended treatment options for DLGG [8]. Our study found that patients who underwent total resection had significantly longer PFS than patients who did not undergo total resection, in both oligodendroglioma and astrocytoma. Ding et al. [9] shows that in oligodendroglioma, resection level has a weak effect on the prognosis, which may be related to the sensitivity of oligodendroglioma to radiotherapy and chemotherapy as well as its growth inertness. Shaw et al. [10] conducted a retrospective study on postoperative MRI of patients under the age of 40 who underwent neurosurgery. It was found that when the residual tumor was less than 1 cm, the recurrence rate was 26% within 5 years; when the residual tumor was 1–2 cm, the recurrence rate was 68% within 5 years, and when the residual tumor was larger than 2 cm, the recurrence rate was 89% within 5 years. The risk of malignant transformation in DLGG is highly dependent on tumor volume and growth rate [11]. In our study, univariate analysis showed significant difference in preoperative tumor volume between the two groups, The tumor volume is larger in the early progression group. Therefore, for occasional or asymptomatic low-grade gliomas, compared to surgery after tumor enlargement, we recommend early surgery. We found that the location of the tumor and the involved range of lobes were closely related to the degree of surgical resection, and it was more difficult to completely remove the deeper tumor involving multiple lobes.

Since tumor is diffusely infiltrating, total resection is usually unrealistic. Even if total resection is performed, risk of progression within 5 years is above 50% [10]. There fore, adjuvant radiotherapy or chemotherapy is often required after surgery. However, the timing of adjuvant treatment is still controversial [12]. Current research supports early postoperative use of radiotherapy and chemotherapy in patients with a greater risk of tumor recurrence [13]. However, for low-risk patients, radiotherapy and chemotherapy are controversial; this is due to that the relationship between postoperative radiotherapy and chemotherapy and malignant transformation has not yet been clarified so far [14]. Previous studies have shown that postoperative radiotherapy can significantly extend progression-free survival. For high-risk patients, the combined application of radiotherapy and chemotherapy can significantly improve progression-free survival and overall survival compared with using radiotherapy alone [13]. In this study, radiotherapy and chemotherapy were performed in the early postoperative period, with no cases of delayed chemoradiotherapy. There was no significant difference in postoperative chemoradiotherapy between the early progression group and the control group, which may be related to the small number of cases in our study. Moreover, cases receiving postoperative radiotherapy and chemotherapy accounted for the majority in our study. Therefore, further studies investigating the effect of adjuvant chemoradiotherapy on the early progression of low-grade glioma are needed.

Our results show that astrocytoma is a risk factor for DLGG progression, and the progression-free survival time of astrocytoma is significantly shorter than that of oligodendrocytoma. Maarten et al. [15] also found that the effect of postoperative residual tumor volume on survival was more pronounced in astrocytomas than in oligodendroglioma, and even tumor with few residue would produce negative effect on overall survival of astrocytoma. However, such effect is not so obvious in oligodendroglioma. In this study, due to the retrospective analysis of the cases before 2015, only part of the cases accepted molecular detection, and the molecular characteristics were not included in the analysis, which is the shortcoming of this study. With the development of pathology and advances in molecular detection technology, more and more molecular markers have been shown to play an important role in glioma typing, grading, treatment, and prognosis; in addition to the currently recognized IDH, 1p/19q molecular state can predict the prognosis of DLGG, and we know very little about the molecular mechanism of rapid progression of low-grade diffuse gliomas. We believe that molecular differences may be an important cause of survival heterogeneity in low-grade diffuse gliomas. Therefore, in-depth molecular analysis of DLGG is needed to identify genetic markers for early or delayed tumor progression.

## Conclusions

The goals of DLGG treatment include prolonging progression-free survival and improving overall survival while maintaining quality of life. Our results show that in adult low-grade diffuse gliomas, astrocytoma is more likely to progress early. The location and incomplete resection of the tumor are factors that affect early progression. When the tumor invades deep location such as basal ganglia, inner capsule, and corpus callosum, it is more difficult to completely remove the tumor and is more likely to progress early. Therefore, for such patients, surgical resection within the greatest possible safety range and more detailed follow-up are required.

## Data Availability

Data were available upon appropriate request.
